# Alterations of Chromatin Regulators in the Pathogenesis of Urinary Bladder Urothelial Carcinoma

**DOI:** 10.3390/cancers13236040

**Published:** 2021-11-30

**Authors:** Michèle J. Hoffmann, Wolfgang A. Schulz

**Affiliations:** Department of Urology, Medical Faculty, Heinrich Heine University Düsseldorf, 40225 Düsseldorf, Germany; michele.hoffmann@hhu.de

**Keywords:** urothelial carcinoma, bladder cancer, chromatin regulator, epigenetic enzyme, differentiation, plasticity, clonal expansion

## Abstract

**Simple Summary:**

Urinary bladder cancer is one of the ten major cancers worldwide, with higher incidences in males, in smokers, and in highly industrialized countries. New therapies beyond cytotoxic chemotherapy are urgently needed to improve treatment of these tumors. A better understanding of the mechanisms underlying their development may help in this regard. Recently, it was discovered that a group of proteins regulating the state of chromatin and thus gene expression is exceptionally and frequently affected by gene mutations in bladder cancers. Altered function of these mutated chromatin regulators must therefore be fundamental in their development, but how and why is poorly understood. Here we review the current knowledge on changes in chromatin regulators and discuss their possible consequences for bladder cancer development and options for new therapies.

**Abstract:**

Urothelial carcinoma (UC) is the most frequent histological type of cancer in the urinary bladder. Genomic changes in UC activate MAPK and PI3K/AKT signal transduction pathways, which increase cell proliferation and survival, interfere with cell cycle and checkpoint control, and prevent senescence. A more recently discovered additional category of genetic changes in UC affects chromatin regulators, including histone-modifying enzymes (KMT2C, KMT2D, KDM6A, EZH2), transcription cofactors (CREBBP, EP300), and components of the chromatin remodeling complex SWI/SNF (ARID1A, SMARCA4). It is not yet well understood how these changes contribute to the development and progression of UC. Therefore, we review here the emerging knowledge on genomic and gene expression alterations of chromatin regulators and their consequences for cell differentiation, cellular plasticity, and clonal expansion during UC pathogenesis. Our analysis identifies additional relevant chromatin regulators and suggests a model for urothelial carcinogenesis as a basis for further mechanistic studies and targeted therapy development.

## 1. Introduction

### 1.1. Genomic Alterations in Urothelial Carcinoma

Urinary bladder cancer is one of the ten major cancers worldwide with a higher incidence in males, in smokers, and in highly industrialized countries [1]. The predominant histological subtype is urothelial carcinoma (UC), with squamous cell carcinoma and further rarer entities making up less than 10% of the cases in most populations. UC comprises two large subgroups, distinguished by whether they have invaded the underlying connective tissue layers of the bladder. Non-invasive papillary UC represents most of the cases. Low-grade papillary UC is not life-threatening, but requires therapy and long-term follow-up. High-grade papillary UC and especially carcinoma in situ have a higher risk of progressing to invasive carcinomas. Of note, tumors of various stages and grades may develop not only metachronously but also synchronously. Invasive carcinomas comprise about one-quarter of the cases at presentation and require radical surgery, often combined with neo-adjuvant or adjuvant chemotherapy. Metastatic UC remains invariably lethal, despite systemic chemotherapy and more recently introduced immunotherapies [2].

Genomic changes in UC alter three regulatory systems in a rather consistent manner (reviewed in [3,4,5]). (1) The MAPK and PI3K/AKT signal transduction pathways that direct cell proliferation and survival are activated by mutations, amplification, or rearrangements of receptor tyrosine kinase genes, most frequently *FGFR3*, by oncogenic *RAS* mutations, by alternate *PIK3CA* oncogenic mutations or *PTEN* loss, or by other mutations in MAPK and PI3K/AKT pathway components. (2) Cell cycle and checkpoint control are disturbed by *TP53* inactivation as well as *RB1* mutations and deletions, which are both much more frequent in invasive than in papillary UC, or by homozygous deletion, promoter hypermethylation, and mutations in *CDKN2A,* encoding regulators of both TP53 and RB1, which are observed across all stages of the disease. Additional relevant changes in cell cycle control include inactivating mutations in *CDKN1A* (encoding p21^CIP1^) and *ATM* as well as amplification of *CCND1* encoding Cyclin D1. (3) Loss of function of TP53 and RB1 is expected to impede the establishment of senescence in response to replication stress and genomic instability. In addition, telomerase is activated in almost all UC, independent of grade and stage, most frequently by activating mutations in the *hTERT* promoter.

Collectively, these changes would have been considered sufficient to explain the pathogenesis of urothelial cancers. It came therefore as a surprise when mutations in chromatin regulator genes were discovered to represent another consistent alteration in UC [6,7]. The vast majority of UC cases contain mutations in at least one of these genes (see Section 2). In particular, mutations in chromatin regulator genes are even found regularly in papillary UC, which often remain near-diploid and have a comparatively low number of genomic changes. UC thus presents with one of the highest frequencies of genetic alterations in chromatin regulator genes among all cancer types. Obviously, altered function of chromatin regulators must be fundamental for the pathogenesis of UC. Why this is so is, however, poorly understood to date.

In the present review, we will therefore attempt to delineate the scope of the alterations in chromatin regulators in UC (Section 2) and to outline possible functional explanations for their high prevalence (Section 3).

### 1.2. Urothelial Differentiation and Molecular Subtypes of Urothelial Carcinoma

The source of urothelial carcinoma is the urothelium, a specialized, stratified epithelium lining the urinary tract from the renal pelvis into the urethra. UC can manifest in any section of the urothelium, but most tumors develop in the bladder. Of note, UC of the upper urinary tract presents with overall similar genomic alterations, albeit at different frequencies [8,9]. Moreover, tumors deficient in DNA mismatch repair occur exclusively in the upper urinary tract.

The adult urothelium is usually quiescent, with one of the slowest turnover rates among mammalian epithelia. However, following injury, its cells are capable of resuming proliferation when rapid regeneration is needed, e.g., after mechanical injury or bacterial infection, allowing complete restoration within 72 h [10,11,12].

The urothelium comprises three different layers of cells with increasing morphological complexity and differentiation: basal cells, intermediate cells, and the superficial umbrella cells that form the major barrier towards the urine in the bladder lumen (reviewed in [13]). The basal cells are attached to the basement membrane and are small with condensed nuclei, in keeping with their rather undifferentiated state. They express basal cell marker proteins like cytokeratin 5 (KRT5) and TP63, but not the specific marker proteins of urothelial differentiation like cytokeratins 7, 8, 18, and 20 or uroplakins. A subset of basal cells (estimated variously as 0.5–10%) also expresses KRT14. Under homeostatic conditions, these may be the only mitotically active cells with some stem cell characteristics [14]. Cells in the rat bladder retaining labeling for more than one year also reside in the basal layer [15]. They may therefore constitute progenitor cells at the origin of clonal units, maintaining and regenerating the urothelium [16], although this interpretation needs further investigation [17].

Intermediate cells, in keeping with their designation, have an intermediate degree of characteristics and express KRT7, little if any KRT5, and no KRT14. At least a subset of intermediate cells can proliferate during acute injury and contribute to regeneration of the urothelial layer, whereas the response to chronic injury depends more on basal cells [17,18,19].

The luminal superficial umbrella cells are larger, multinucleated, and terminally differentiated. They express uroplakins, specialized proteins required for the barrier function of the urothelium, and specifically KRT20. To accommodate varying filling volumes in the bladder, the thickness of the epithelium can adapt by intermediate cells shifting against each other, and umbrella cells can adjust their apical surface area [20].

Urothelial regeneration following injury is stimulated by several growth factors from urothelial cells and mesenchymal cells in the underlying submucosal connective tissue. Injured urothelium secretes EGF-like growth factors (such as TGFα, HB-EGF, and amphiregulin) to stimulate proliferation. Signaling through FGF receptors activated by FGF2 contributes to this process. Reciprocal epithelial-mesenchymal signaling, with basal cells secreting SHH to stimulate WNT secretion in mesenchymal cells, is likewise activated during rapid regeneration [21,22].

Differentiation in the urothelium is regulated by a network of transcription factors, which is still incompletely understood [23,24]. These transcription factors are thought to interact with various chromatin regulators to establish stable and adaptive transcription patterns, but few studies have addressed these interactions in the urothelium. However, since the urothelial transcription factor network bears similarities with the networks regulating epidermal and ductal mammary differentiation, observations from these tissues may extend to the urothelium (see below).

The *TP63* gene encodes various isoforms of a p53 family transcription factor. The most prominent isoforms are the full length variant TAp63 and the N-terminal truncated ΔNp63 isoform with opposing functions in the regulation of differentiation and proliferation that carry over into tumors with a basal cell phenotype. The ΔNp63 variant is the most strongly expressed isoform in the basal layer of stratified epithelia. It maintains the proliferation potential of progenitor cells and controls epithelial morphogenesis through recruitment of epigenetic regulators, including repressive complexes containing histone deacetylases (HDAC) [10,25]. ΔNp63 can in particular interact with the SWI/SNF chromatin remodeling complex.

The transcription factor ZNF750 controls epithelial tissue homeostasis by repression of progenitor genes and upregulation of differentiation genes through interaction with the transcription factor KLF4 and chromatin regulators like KDM1A and HDAC1 [26]. ZNF750 expression is in turn regulated by TP63 [27]. The Grainyhead transcription factor GRHL3 interacts with other chromatin regulators, including JMJD3 (*KDM6B*) and BRG1/BRM (*SMARCA4/A2*) and the histone methyltransferase MLL2 (*KMT2D*) [28]. These factors have been investigated in more detail for their function in epidermal differentiation but are also implicated in urothelial homeostasis [29].

The nuclear receptor PPARγ has been shown to promote specifically urothelial over squamous differentiation. In mice, *Pparg* mutations prevented maturation of superficial umbrella cells, and *Pparg* inactivation in basal cells resulted in squamous rather than urothelial differentiation. Further, *Pparg* mutant mice presented with persistent inflammation in the urinary tract and activated NFκB signaling. Accordingly, Pparg re-expression prevents squamous differentiation [30]. Additional transcription factors like Elf3, Grhl3, and Klf5 and specific Gata factors were identified as determinants of urothelial specification in mice [25,31,32,33].

To investigate the transcription factors involved in human urothelial differentiation, a model system using cultures of normal human urothelial (NHU) cells has been employed [34]. These cells, usually isolated from the ureter or, less commonly, the bladder urothelium, proliferate in specific serum-free media under the influence of EGF-like and FGF factors [35]. Notably, they acquire a basal cell phenotype with increased plasticity, and a substantial fraction of the cells express the stem cell marker KRT14. Accordingly, they can be induced by different protocols towards squamous or urothelial differentiation.

Induction of urothelial differentiation in this model system is achieved by blocking EGFR signaling and activating PPARγ. This leads to the expression of intermediate transcription factors specifying the differentiated urothelial cell phenotype, including forkhead box A1 (FOXA1), interferon regulatory factor 1 (IRF1), GATA-binding protein 3 (GATA3), and E74-like ETS transcription factor 3 (ELF3) [36]. Concomitantly, the cells acquire markers of luminal urothelial differentiation like KRT20 and uroplakins. An alternative protocol for differentiation employs treatment with serum and calcium with similar consequences but more pronounced stratification. Thus, a network of transcription factors, including PPARγ, IRF1, FOXA1, ELF3, and GATA3, lies at the core of urothelial differentiation [29].

Reflecting their increased plasticity, a different protocol applying high dose calcium only results in the stratification and squamous differentiation of primary urothelial cells. This state is characterized by expression of KRT5/6, KRT14, and S100A9 and further epidermal differentiation markers like Loricrin, Filaggrin, and Involucrin [37]. Of note, this protocol can also be successfully applied to HBLAK cells, a non-transformed urothelial cell line [38].

Characteristics of basal and luminal differentiation states are reflected in the recently defined molecular subtypes of urothelial carcinoma. The recent consensus classifies invasive urothelial carcinomas into six subclasses, mainly on the basis of gene expression patterns [39]. The subclasses differ, moreover, by their predominant driver mutations, prognosis, and response to therapy, albeit with substantial overlaps.

Luminal subtypes are characterized by the expression of markers of urothelial differentiation, including uroplakins and KRT20, which reflect the activity of the transcription factors FOXA1, GATA3, and PPARγ. This applies in particular to the luminal papillary subtype characterized by oncogenic mutations activating the growth factor receptor FGFR3 and *CDKN2A* deletions. In contrast, mutations in *TP53* (and *RB1*) are relatively rare in this subtype. The frequency of mutations in the *KDM6A* histone demethylase gene is likewise highest in this subtype. Importantly, this subtype resembles the predominant molecular subtype of non-invasive papillary urothelial tumors [40]. Luminal papillary cancers have the best prognosis of all invasive UCs, and even metastatic cases often respond well to treatment with recently introduced FGFR3 inhibitor drugs (reviewed in [41,42]).

The second luminal subtype (luminal unstable) contains the largest number of genomic changes and displays the highest cell cycle activity of all subtypes. *TP53* mutations and *ERBB2* amplification are prevalent in this subtype, but also mutations and gene fusions activating the lineage-specific oncogenes *PPARG* and *RXRA* (encoding the standard PPARγ heterodimerization partner) [43].

A third, rarer luminal subtype (luminal non-specified) contains more stromal cells, especially fibroblasts. Mutations in the *ELF3* gene, which encodes a transcription factor acting downstream of PPARγ in urothelial differentiation [32], are enriched in this subtype. Of note, FGFR3 mutations are less common in the luminal unstable and luminal non-specified subtypes than in luminal papillary cancers [7,39].

Stromal-rich tumors contain an even higher fraction of various stromal cell types, but additionally of immune cells. Urothelial differentiation markers are less prominently expressed.

The basal-squamous subtype (BASQ) is characterized by the expression of differentiation markers of basal urothelial cells (see above) and a variable expression of markers of squamous differentiation. Regions with histological squamous differentiation are detectable in up to 40% of these tumors. Gene expression patterns indicate active EGFR and STAT3 signaling. *TP53* and *RB1* mutations are frequent. BASQ tumors often contain many immune cells, including cytotoxic T-cells.

Neuroendocrine-like cancers constitute the rarest subtype. Most are morphologically identifiable as ‘small cell carcinomas’, while others can only be assigned to this subtype by their expression patterns. Typical of neuroendocrine-like cancers, *TP53* and *RB1* are concomitantly inactivated. Neuroendocrine-like cancers have the worst prognosis of all UC subtypes, followed by BASQ tumors [39].

Squamous cell carcinoma (SCC) of the bladder is regarded as a distinct histologic entity from urothelial carcinoma. These rarer cancers usually arise following chronic inflammation of the bladder, caused, e.g., by Schistosoma infection or catherization. Genomic alterations in SCC are however similar to those in BASQ UC, suggesting that they arise by a similar mechanism. Presumably, aberrant urothelial regeneration during chronic inflammation results in squamous metaplasia that can progress to SCC [10]. The plasticity of urothelial precursor cells in vitro (see above) supports this contention.

### 1.3. Chromatin Regulators

Regulation of the chromatin structure is required for transcription regulation, genome replication, mitosis, DNA repair, formation of constitutive heterochromatin, and other nuclear processes. Cellular plasticity, lineage choices, and cell differentiation are controlled by dynamic epigenetic mechanisms. At the core of epigenetic regulation, establishment, maintenance, and changes of cell differentiation states are associated with chromatin states, delineating the basic transcription patterns of each cell type. These long-term stable chromatin configurations are then dynamically adapted to various functional requirements, including cell replication, specific cell function, metabolic demands and various stresses (reviewed in [44,45,46,47]).

Chromatin regulators encompass a variety of factors acting on DNA, histones, and other components of chromatin and interacting among themselves. DNA methylation at cytosines in CpG-dinucleotides is a largely long-term modification that is established by DNA methyltransferases (the ‘writers’) and removed by DNA demethylases (the ‘erasers’) and additional mechanisms, such as ‘passive’ dilution by replication without remethylation (reviewed in [48]). It is recognized positively (‘read’) by proteins like MBD2, which typically act as components of repressor complexes, but cytosine methylation also prevents binding of transcriptional activators to CpG-containing binding sites, to very different degrees (reviewed in [49]). Additionally, DNA methyltransferases and demethylases interact directly with other types of chromatin regulators. Alterations of DNA methylation patterns are frequent in UC. Pathogenesis and functional consequences of these alterations and their use in bladder cancer diagnostics have been reviewed elsewhere [50,51,52,53,54].

Histone modifications mediate both stable and dynamic chromatin states. For instance, heterochromatin, active and inactive enhancers, active and repressed genes, promoters, and gene bodies are each distinguished by patterns of specific histone modifications, including lysine and arginine methylation and lysine acetylation. Phosphorylation of histones is particularly relevant for DNA repair and mitosis. Overall, more than 200 different histone modifications are known (listed in [55]), and their number is still increasing. Like DNA methylation, histone modifications involve writers and erasers and are interpreted by readers, which include many proteins that also act as writers or erasers, allowing forward and feedback regulatory circuits. 

Acetylation of different lysine residues, especially in the N-terminal non-helical domains of histones H3 and H4, is generally a marker of open chromatin and in particular associated with actively transcribed genes and active enhancers. Histone acetylation is regulated by a dynamic interplay of histone acetyltransferases (HATs) and histone deacetylases (HDACs). Many HATs are transcriptional co-activators, like p300 (gene *EP300*) and CBP (gene: *CREBBP*). Notably, like these two proteins, many HATs have paralogs with overlapping but not identical functions, like the pairs GCN5/PCAF (*KAT2A/KAT2B*) and MYST3/MYST4 (also known as MOZ/MORF, encoded by *KAT6A/KAT6B*). In its function as a co-activator, each HAT interacts with a set of cell-type-specific or ubiquitous transcription factors to establish and maintain open chromatin structures at enhancers (especially p300) and promoters. Other HATs function mostly in the deposition of newly formed nucleosomes following DNA replication or in DNA repair as components of multiprotein complexes. Histone acetylation is removed by hydrolysis catalyzed by histone deacetylases (HDACs). Among the 18 HDACs in human cells, subclass I enzymes (comprising HDAC1, 2, 3, and 8) catalyze the bulk of histone deacetylation. However, HDACs have non-histone protein substrates as well, and some are located predominantly in the cytoplasm or shuttle between cytoplasm and nucleus (like subclass IIA enzymes HDAC4, 5, 7, and 9). Acetylated histones are specifically recognized by a large number of nuclear proteins containing PHD domains or bromodomains (reviewed in [56]).

The function of lysine methylation depends on the site and the number of methyl groups. Histone methylation is in general less dynamic than acetylation. For instance, trimethylation of lysine 9 (H3K9me3) is characteristic of constitutive heterochromatin, whereas H3K4me3 marks active promoters. H3K27me3 is a repressive modification found especially at genes regulated during development, lineage choice, or cell differentiation. Methylation at H3K36 distinguishes actively transcribed gene bodies. In a similar fashion, methylation at various sites of other canonical and of variant histones (such as H3.3, H2AZ, and H2AX) is associated with different chromatin states. Accordingly, different histone methylases from several enzyme and gene families attach methyl groups, and conversely, histone demethylases with various specificities remove them. Most histone demethylases, with KDM1A as a notable exception, are dioxygenases (like the TET family of DNA demethylases) and use α-ketoglutarate as a co-substrate. In the context of UC, the KMT2 family and EZH2 have received most attention. The KMT2 H3K4 histone methylases (also known as MLLs) are components of multiprotein complexes that activate transcription at promoters and enhancers (reviewed in [57]). EZH2 is the catalytic subunit of the polycomb repressor complex PRC2 (which may alternatively contain its paralog EZH1), which mediates H3K27 trimethylation and gene repression. Removal of methyl groups at H3K27 is initiated by the KDM6 demethylases KDM6A (also known as UTX) and KDM6B (also known as JMJD3). The third family member, UTY, has little enzymatic activity, but can substitute for KDM6A in some contexts [58,59]. 

Chromatin remodelers form another class of chromatin regulators. They interact with histone-modifying proteins and DNA-binding transcription factors to establish and change overall chromatin structure. In particular, several multiprotein complexes move nucleosomes along DNA, thereby creating denser packaging or more open structures. Chromatin remodelers like the SWI/SNF complex are therefore required for both gene repression and activation, but remodelers are also necessary for DNA repair and replication or for exchange of histone variants (like the SWR1 complex). In the context of cancer, the SWI/SNF complex has received most attention, as several of its components are mutated at significant frequencies in many cancer types, including UC (reviewed in [60,61,62]). The best recognized function of this complex is the regulation of gene activation at transcriptional start sites, where repositioning of nucleosomes facilitates the access of transcriptional activators and the basal transcription machinery. The SWI/SNF complex is also notable for interacting with many nuclear receptors, including the estrogen receptor, androgen receptor, and PPARγ. Nucleosome repositioning by SWI/SNF is driven by ATP through the ATPases BRG1 (gene *SMARCA4*) or BRM (gene *SMARCA2*) in different isoforms of the complex. ARID1A and ARID1B are further essential components present in alternative isoforms. ARID1A is particularly frequently inactivated in cancer [63,64]. Helicase activity is provided by the HELLS subunit (also known as LSH, gene *SMARCA6*). A number of chromodomain protein (CHD) subunits are named for a domain recognizing methylated histones. 

At a higher level of organization, chromatin looping defines local segments along the DNA strand, within which enhancers and promoters can interact. It is organized among other factors by the DNA-binding ‘isolator’ protein CTCF and by the cohesin complex (reviewed in [65,66]). One cohesin component, STAG2, is inactivated by mutations in up to 20% of UC [7,67,68]. This inactivation was initially assumed to contribute to the pronounced chromosomal instability seen in many invasive UC. However, no consistent correlation with the degree of chromosomal instability could be confirmed [67,69], and recent findings in other cancer types suggest an impact on transcription regulation through chromatin organization [70,71]. Of note, *STAG2* and another frequently mutated chromatin regulator gene, *KDM6A*, are located on the X-chromosome, which may contribute to the overall male gender bias of UC [40].

Histone variants provide another option for chromatin regulation; they mark and support the function of specific genome regions such as centromeres, telomeres, active genes, and regions involved in nucleolar functions. H2AX is essential for DNA repair. Only a few studies to date have addressed the function of histone variants in UC [72,73]. As it is still difficult to assess their impact, we will not deal further with histone variants in the present review. Rather, we will primarily consider histone-modifiers (including erasers) and chromatin remodelers as core chromatin regulators (see also introductory remarks to Section 2).

In addition, various types of non-coding RNAs are involved in the regulation of chromatin states and gene expression. Long non-coding RNAs act at many steps of gene expression regulation, and several are involved in the establishment and maintenance of chromatin structures (reviewed in [74]). Altered expression of several lncRNAs has been detected and confirmed in UC, as reviewed elsewhere. Micro-RNAs are primarily post-transcriptional regulators influencing chromatin states only indirectly. Both overexpression and down-regulation of specific miRNAs has been reported in UC, and the functions of these changes have been widely studied, although not all reports are consistent. Several recent reviews on expression and functions of miRNAs in UC are available [75,76]. Of note, with further research and validation of preliminary studies, lncRNAs or miRNAs may provide particularly valuable biomarkers in UC diagnostics.

## 2. Alterations of Chromatin Regulator Genes in Urothelial Carcinoma

### 2.1. Introductory Remarks

Several studies have consistently revealed that chromatin regulators, specifically chromatin remodelers and histone-modifying enzymes, are exceptionally frequently mutated in UC [6,7,68,77], suggesting that subsequent epigenetic changes drive urothelial tumorigenesis and cancer progression (reviewed in [78,79]). In the TCGA study on muscle-invasive UC [7], about 90% of the cancers carried one or more mutations in *KDM6A* (encoding histone demethylase UTX), *KMT2C*, *KMT2D* (encoding histone methyltransferases MLL2 and MLL3), *CREBBP*, *EP300* (histone acetyltransferases), and *ARID1A* (a SWI/SNF component).

To obtain a comprehensive overview of genomic alterations affecting chromatin regulators in UC, we downloaded a list of 720 genes categorized as chromatin regulators in the EpiFactors database, a manually curated database for epigenetic regulators and their complexes [80]. Note that the definition of epigenetic regulators in the Epifactors database is somewhat broader than that outlined in Section 1.3 by including selected transcription and RNA-processing factors and DNA methylation writers and erasers. In order to avoid introducing bias, we did not perform an annotation prior to the following analysis. All 720 genes were searched for mutations, copy number alterations (CNA, restricted to amplifications or homozygous deletions), and expression differences in bladder cancer tissues in comparison to normal bladder tissues in the TCGA dataset (BLCA). To this end, public TCGA expression data were downloaded using the Morpheus tool generated by the Broad Institute (https://software.broadinstitute.org/Morpheus, accessed on 31 August 2021). Expression values for the 720 epigenetic regulators were extracted for tumor and normal samples, and fold-change expression across tumor samples as compared to normal samples was calculated. Data on mutations and genetic changes in the TCGA BLCA dataset were obtained from Robertson et al. [7] via BioPortal [81,82] and filtered for results on the 720 chromatin regulators. 

### 2.2. Mutations

Among the 720 chromatin regulator genes, 665 contained nonsynonymous mutations in at least one of the 412 UC samples; 187 were mutated in at least 10 tumors. Table 1 lists the top 25 most frequently mutated genes; a complete listing can be found in the Supplementary Materials (Data file S1). As reported by the TCGA authors [7], the most frequently mutated genes were the COMPASS components *KMT2C*, *KMT2D,* and *KDM6A*, and the SWI/SNF subunit *ARID1A*, along with *TP53* (a transcription factor), each in more than 100 tumors. Genes mutated in at least 10 tumors encoded many additional histone writers, erasers, and components of chromatin remodeling and polycomb complexes, as well as a few transcription factors, coactivators, and corepressors, including *FOXA1* in 14 cases, *PPARGC1A* (a PPARγ coactivator) in 19 cases, and *RB1* (a corepressor) in 83 cases. Each of the three TET DNA demethylase genes was mutated in at least 10 cancers, most frequently *TET1*, in 30 cases. *DNMT3A* and *DNMT1* were each affected by mutations in 10 cases. This latter result suggests that DNA methylation changes in UC [50,51,52,53,54] may in some cases be caused by genetic alterations of DNA methylation writers and erasers.

These numbers have to be considered in the light of the high mutation rate in UC (up to 10/Mbp), with 100–200 genes mutated in typical cases. In addition, our present analysis does not distinguish the type of mutation in the respective genes, such as missense, nonsense, or other types of truncating mutations. In evaluating the significance of mutations, the ratio of nonsynonymous to synonymous mutations would also have to be considered for each individual gene (see ref. [7], supplement). Nevertheless, our review indicates that various chromatin regulators are affected by mutations in individual UC, in addition to those formally identified as significantly mutated genes in the overall TCGA cohort, such as *KMT2C*, *KMT2D*, *KDM6A, ARID1A,* and others.

A total of 438 chromatin regulator genes were amplified in at least one UC, with 147 loci amplified in at least 10 cancers. Table 2 lists the top 25 most frequently amplified genes; a complete listing can be found in the Supplementary Materials (Data file S1). Amplified regions harboring more than one chromatin regulator gene were predominantly located on chromosome arms known to be gained in UC [4,83] and included especially 1p, 8q, 5p, and 10p (which are therefore over-represented in Table 2) as well as at lower frequencies 1q, 3p(distal)/3q, 6p (the well-described 6p22 amplicon [84] with the *DEK* gene [85]), 11q, 16p, 17q, 19, and 20q. Interestingly, the most frequently amplified chromatin regulator gene was *USP21* at 1q23.3, which encodes a H2A deubiquitinase. The enzyme furthermore acts on a large number of non-histone proteins, including EZH2 [86] and BRCA2 [87]. While investigated in other cancers, few studies on the function of USP21 in UC are available to date. In one study, the 1q23 amplicon has been described as being associated with metastasis and poor prognosis [88]. Another amplicon at 1q21.3 contains *SETDB1* as previously highlighted by others [89]. The encoded H3K9 methyltransferase is essential for the growth of many cancer types and considered as a therapeutic target [90]. Its function in UC is however unexplored. At 8q22.3, *YWHAZ* and *UBR5* were frequently amplified. *YWHAZ* encodes a 14-3-3 family protein with multiple functions that is overexpressed due to amplification in different cancer types, including bladder cancer [91]. The E3 ubiquitin ligase UBR5 is likewise overexpressed in different cancers due to gene amplification [92] but has not yet been studied explicitly in bladder cancer. Among its various functions is regulating ubiquitination of chromatin during DNA repair [93]. Whether these two factors impinge on UC pathogenesis as chromatin regulators or through their various other activities is unknown. Other amplified genes of interest are discussed in subsequent sections.

A total of 225 chromatin regulator genes were homozygous deleted in at least one UC, with 39 homozygous deleted in at least 10 cases. Table 3 lists the top 25 most frequently homozygous deleted genes; a complete listing can be found in the Supplementary Materials (Data file S1). Homozygous deletions were predominantly located on chromosomal arms known to be subject to losses in UC [4,83,94]. Several genes each were affected on 2q, 8p21, 9p24 (including *JAK2*, doubling as a histone kinase in addition to its function in STAT signaling), 13q (two distinct regions encompassing *BRCA2* or *FOXO1* and *RB1*, respectively), 16p (including *CREBBP*), 17p (two regions encompassing *NCOR1* and *TP53*, respectively), and 22q13 (encompassing *BRD1* and *HDAC10*). Homozygous deletions at 3p, 4q, 14q, and 18q each contained only a single chromatin regulator gene, namely *FOXP1*, *ING2*, *RCOR1,* and *ZNF516*, respectively. *RB1* was most frequently subject to homozygous deletions in 37 cancers, followed by three genes on 8p.

In addition to those genes discussed in detail in the following subsection, several are good candidates for further studies. *NCOR1* was identified as a significantly mutated gene by several studies [6]. It encodes a co-repressor interacting with many nuclear receptors, influencing especially metabolism and immune responses. Experimental modulation of NCOR1 has been shown to affect PPARγ signaling in prostate and UC cancer cells [95,96], but the relevance of this interaction for UC development and progression remains to be further elucidated. RCOR1 is best characterized as a component of another corepressor complex, Co-REST, which is involved in lineage choice and differentiation. No specific studies on urothelial cells are published, but intriguingly, RCOR1 is required in epidermal progenitor (basal) cells [26]. ZNF516 likewise interacts with CoREST [97], especially to repress EGFR (see also below). In contrast, BRD1 is a bromodomain-containing co-activator interacting especially with the HBO1 complex containing MYST3 (*KAT2A*) to acetylate histones [98]. No dedicated investigations on BRD1 in UC are available. In addition to FOXA1, several other members of the FOX family are implicated in UC (reviewed in [24]), but FOXP1 and FOXO1 (see also below) are not well studied. HDAC10 is a class IIB histone deacetylase that is weakly expressed in urothelial cells [99]. The ING2 protein contributes to nucleotide excision repair and cell cycle control in a TP53-dependent manner; polymorphisms in the gene may influence bladder cancer risk [100].

### 2.3. Expression Changes

Employing a twofold change cut-off, 58 genes were identified as down-regulated in UC compared to normal tissues, whereas 50 were upregulated. These genes are comprehensively listed in the Supplementary Materials (Data file S1).

**Figure 1:**
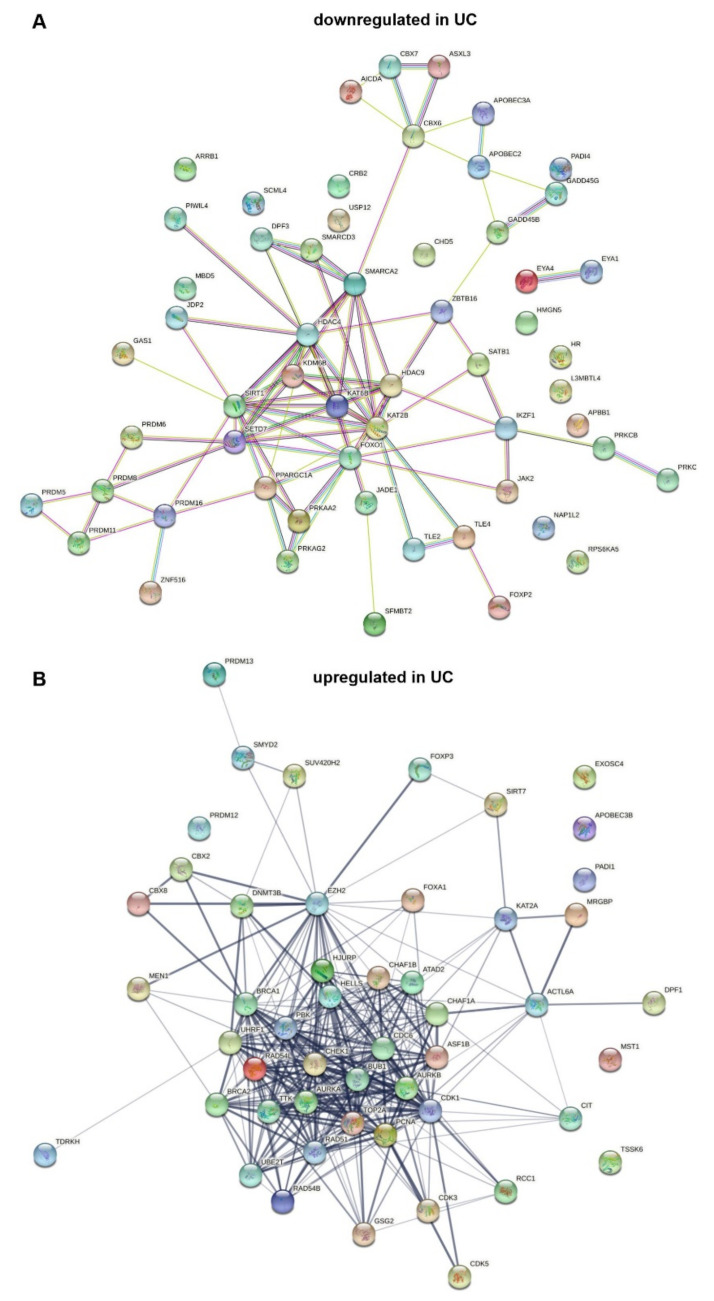
String analysis of chromatin regulator genes downregulated (**A**) and upregulated (**B**) in urothelial carcinoma.

String analysis [101] of downregulated genes (Figure 1A) reveals a highly linked core network containing the HATs *KAT6B* (MYST4, MORF) and *KAT2B* (PCAF), class IIA HDACs 4 and 9, and the class III (sirtuin) HDAC *SIRT1*, the *KDM6B* demethylase, as well as components of SWI/SNF, namely *SMARCA2*/BRM, *SMARCD3*/B60c, and *DPF3*/BAF45c. Notably, other components of the SWI/SNF multiprotein complex are upregulated (Figure 1B), hinting at a shift in its composition. Three components of the PRC1 polycomb complex are distantly linked to the core network. Two important transcription factors are highlighted by the analysis. *PPARGC1A* encodes a co-activator of PPARγ, a key regulator of urothelial differentiation (see Section 1). FOXO1 downregulation has been implicated as an important factor in the progression of UC and in resistance to chemotherapy by several studies [102,103,104]. The present analysis suggests that its downregulation may be complemented by downregulation of co-regulators, including HAT coactivators.

The *SETD7* H3K4 monomethyltransferase connects the core network with several *PRDM* genes. These encode proteins that likewise function as histone methylases or regulate such enzymes. PRDM proteins have been shown to exert tumor-suppressive or oncogenic functions in different cancer types (reviewed in [105]); *PRDM12* and *PRDM13*, interestingly, are upregulated (see Figure 1B). No dedicated investigation on any family member in bladder cancer has been published, but previous bioinformatic analyses by others have also highlighted these factors [105].

String analysis of upregulated genes yields an entirely different core network, dominated by histone kinases associated with mitosis (*AURKA/B, TTK, GSG2/HASPIN, PBK* and others) and regulators of DNA replication and cell cycle progression (*CDC6, CDK1, CDK3, CDK5, TOP2A*) and DNA methylation (*DNMT3B, UHRF1*). Like the upregulation of these factors, upregulation of chromatin assembly factors (*CHAF1A/B*, encoding CAF1, and the reader *ATAD2*) may simply reflect the increased proliferation of urothelial cancer cells compared to quiescent normal urothelium. Interestingly, CAF1 is involved particularly in the deposition of variant histones and can influence differentiation and reprogramming in this manner [106,107]. A range of genes encoding proteins involved in homologous recombination DNA repair (*BRCA1/2*, *RAD51*, *UBE2T/FANC2*, *CHEK1*, *PCNA*, *TOP2A*) is also upregulated, possibly reflecting an increased requirement in cancer cells subject to replication stress. The histone methyltransferase *EZH2*, a central component of the PRC2 polycomb complex, is well known to be upregulated in UC [108,109]. Likewise, upregulation of *KAT2A* (GCN5), with concomitant downregulation of its close paralog *KAT2B,* is a common finding in cancer [110]. In UC, few studies on this pair of HATs are available, but GCN5 indeed appears to contribute more to neoplastic properties of UC cells than PCAF [111]. An interesting observation is upregulation of FOXA1, a pioneer forkhead transcription factor involved in urothelial differentiation and mutated in a subset of UC [112,113].

Concordantly with their downregulation, *PPARGC1A*, *ASXL3*, *SIRT1*, *SMARCA2,* and the histone methylase genes *PRDM5/6/16* were also affected by mutations or homozygous deletions in at least 10 tumors. The *ZNF516* gene, encoding a repressor with possible antitumor functions [97], was also subject to homozygous deletions, like the *HDAC4* gene. No specific investigations on the ZNF516 repressor in bladder cancer have been published, whereas several investigations suggest that the class IIA histone deacetylase HDAC4 inhibits neoplastic properties of bladder cancer cells [114,115]. In comparison, the *HDAC9* gene was amplified in a substantial number of cases, but its expression was downregulated on average.

Several of the upregulated genes were also subject to mutations or amplifications in at least 10 tumors. These included the genes encoding topoisomerase TOP2A, mitotic regulator BUB1, DNA methyltransferase DNMT3B, as well as *ACTL6A* encoding an actin component of chromatin remodeling complexes. ACTL6A is reported to contribute to the repression of CDKN1A/p21^CIP1^ in squamous carcinomas [116], but no studies on its function in UC are available. Furthermore, *CDC6*, encoding a chromatin licensing factor upregulated in many cancers with a presumed oncogenic function (reviewed in [117]), was mutated and amplified in many tumors. The few studies on CDC6 in bladder cancer published to date suggest a particular role in cisplatin resistance [118]. Like *CDC6*, *ATAD2* is an E2F1 target upregulated in proliferating cells. Its product, a H4 dual acetylation reader [119], is accordingly overexpressed in many cancer types and considered to contribute to the deregulation of several signal transduction pathways (reviewed in [120]). However, for bladder cancer specifically, no studies on this important factor are available.

A few upregulated genes were—paradoxically—affected by homozygous deletions. This non-concordance may be explained by differences between individual molecular subtypes, especially in the case of *FOXA1*, which directs luminal differentiation. These genes in addition comprise *PBK* (encoding a protein kinase also known as TOPK with a broad spectrum of targets, including histones [121]) and *HJURP*, encoding a chaperone for centromeric variant histones. Intriguingly, a sole publication on bladder cancer so far implicates HJURP in the regulation of PPARγ and SIRT1 [122]. A likewise single study of PBK in bladder cancer demonstrated higher expression in muscle-invasive UC by immunohistochemistry [123] in accordance with the current expression analysis.

*BRCA1* and *BRCA2* were on average upregulated (by 2.8-fold and 3.7-fold, respectively), but a significant number of mutations and—for *BRCA2*—homozygous deletions was found. This likewise suggests heterogeneity among UCs in their capacity for homologous recombination repair (see Section 2.4).

### 2.4. Discussion, including Literature Data

Previous comprehensive analyses of UC genomes [6,7,68] have highlighted the inactivation of proteins associated with gene activation at enhancers required for reprogramming and cell differentiation (reviewed in [124]) among chromatin regulators. These proteins include the histone methyltransferases KMT2C and KMT2D (as well as KMT2B in fewer cases), the histone demethylase KDM6A, components of the SWI/SNF complex (most often ARID1A), and the HATs CBP and p300. Importantly, these factors collaborate and interact with each other, and many are components of the same multiprotein assemblies, like the COMPASS complex, which contains alternatively KMT2C or KMT2D as its core unit and KDM6A as an associated important subunit (reviewed in [125]). Accordingly, these mutations tend to occur in a mutual exclusive manner in UC, suggesting that they lead to similar consequences [79]. Importantly, almost each case of UC carries at least one mutation in one of these genes. Of note, while most mutations in these genes are predicted or demonstrated to be deleterious, it is not established for each of the genes whether both alleles are regularly inactivated in a manner expected for ‘classical’ tumor suppressors (see discussion in [79]).

The group of factors regularly mutated in UC can be considered as trithorax-like activators that counteract the activity of polycomb repressor complexes during development and cell differentiation (reviewed in [126,127]). Basically, two polycomb complexes, PRC1 and PRC2, are distinguished, which can each vary in composition. The present analysis confirms again that EZH2, one of two alternative catalytic subunits of the polycomb repressor complex PRC2, is upregulated in many UCs. Regarding the PRC1 complex, most studies in UC have focused on the BMI1 subunit, which is implicated in promoting invasion by repression of microRNA genes [128] and as a possible stem cell factor. Other PRC1 components are less well studied.

Polycomb complexes are established as key regulators of epidermal differentiation [129]. PRC1 and PRC2 have different roles but cooperate in epidermal precursor cells. PRC2 maintains proliferation of precursor cells, preventing premature differentiation by repressing epidermal specific genes. PRC1 represses alternative lineage choices. During differentiation, however, a differently composed PRC1 complex promotes the expression of epidermal genes, especially for adhesion factors [130]. As our analysis revealed downregulation of several PRC1-related genes, it could be interesting to investigate whether the composition of the PRC1 complex changes in UC.

Gene repression by PRC1 is partly mediated via histone H2A mono-ubiquitination, resulting in the H2AK119ub1 modification. This modification is removed by a protein complex containing the deubiquitinase BAP1 as the central unit. Inactivating mutations of BAP1 and deletion of its gene at 3p are prominent in renal cell carcinoma [131] but have also been described to co-occur with *KDM6A* mutations in UC [132]. Our analysis revealed significant downregulation and frequent mutations of *ASXL3.* ASXL proteins in general serve to link chromatin and chromatin-modifying protein complexes. ASXL3 is less well studied than its paralogs but was shown to promote BAP1 binding to its histone substrate like its paralogs [133]. Its downregulation in UC could therefore ultimately contribute to gene repression.

As suggested by its name, BAP1 (BRCA1-associated protein 1) influences also DNA repair (reviewed in [134]), especially through BRCA1. Our present analysis unexpectedly revealed considerable and significant upregulation of both *BRCA1* and *BRCA2* expression across UC, but also relatively frequent mutations in both genes and even homozygous deletions of *BRCA2*. Inactivation of homologous recombination repair is thought to occur relatively infrequently in bladder cancer and biallelic inactivation of *BRCA1* or *BRCA2* is rare. Mutational signatures reflecting homologous recombination repair deficiency (‘BRCAness’) are found in fewer than 10% of all invasive UCs [135]. The present analysis suggests that, instead, upregulation of *BRCA1* and *BRCA2* is prevalent, potentially in a different subset of cancers. In particular, loss of KMT2C in UC cells was shown to decrease the expression of DNA repair genes. Moreover, Kmt2c knockout in mice induced ureter tumors with a high level of DNA double-strand breaks [136]. This genomic instability was ascribed to a co-activator function of the COMPASS (a.k.a. ASCOM) complex for TP53. UC cells with *KMT2C* mutations were moreover more sensitive to PARP inhibitors like olaparib [137]. However, PARP inhibitors, which exploit deficiencies in homologous recombination repair in tumor cells, have so far shown little benefit as a monotherapy for bladder cancer in clinical trials [138]. As more drugs targeting homologous recombination repair deficiency are becoming available, the state of homologous recombination repair in UC clearly deserves further clarification.

Another interesting factor highlighted by our analysis is the class III histone deacetylase SIRT1. SIRT1 deacetylates H4 but also many non-histone proteins, including TP53, as well as other proteins involved in DNA repair and checkpoints. In some cancer types, SIRT1 is upregulated and therefore considered as a target for specific inhibitors [139]. Pharmacological activators are also available (reviewed in [140]) and may be applied in those cancer types where SIRT1 is instead downregulated. The present analysis revealed downregulation, mutation, and homozygous deletion in many UCs, suggesting that they belong to the latter type. According, a sirtuin activator inhibited bladder cancer growth in organoids [141]. Moreover, SIRT1 was described to interact in a feedback loop with PPARγ [142].

## 3. Consequences of Chromatin Regulator Alterations in Urothelial Carcinoma

### 3.1. Differentiation, Plasticity, and Immune Responses

The trithorax-like factors regularly inactivated by mutations in UC are known to be involved in the differentiation of many cell lineages (reviewed in [44,143]). One would therefore presume that their inactivation in UC would lead to a block in differentiation. However, many UCs present with markers of differentiated urothelial cells, especially all luminal subtypes. Moreover, in the TCGA study, genomic alterations of trithorax-like genes, SWI/SNF genes, and cohesin complex genes were essentially equally frequent across all molecular subtypes (see Figure 3 in ref. [7]).

The luminal-papillary subtype expresses a particularly broad range of urothelial differentiation markers and is characterized especially by frequent *KDM6A* loss of function and *FGFR3* oncogenic mutations. Intriguingly, active FGFR3 leads to repression of many enhancers of luminal urothelial genes that are normally bound and activated by KDM6A [144]. This antagonism explains why the two genetic alterations collaborate in tumorigenesis but not why the ensuing tumors prominently express luminal genes.

The function of the PRC2 catalytic subunit EZH2, a direct antagonist of KDM6A with respect to H3K27 methylation, is intriguing in this context. As in epidermal differentiation [129], EZH2 supports the proliferation of basal cells, while limiting the differentiation of intermediate and superficial cells in the normal murine urothelium. However, its expression prevents squamous differentiation and ectopic expression of Krt14 in mouse models [145]. Global comparisons of epigenetic modifications between human bladder cancer cell lines and normal urothelial cells moreover suggest that EZH2 is involved in regulating the balance between normal differentiation and malignant transformation. Trimethylation at H3K27 was located in a more focal manner at transcriptional start sites in normal cells, whereas the modification was distributed more evenly across gene sequences [146]. This redistribution may reflect the frequent upregulation of EZH2 [109] and loss of the KDM6A demethylase in many UCs. Accordingly, the KDM6A paralog KDM6B/JMJD3 is known to contribute to differentiation of cultured primary keratinocytes [147]. In primary urothelial and HBLAK cells, knockdown of KDM6A affected the balance between KRT14-positive stem cells and KRT5-positive basal cells but had only limited effects on further differentiation of luminal cells [148].

Other luminal subtypes appear to be supported by lineage-specific oncogenes, i.e., transcription factors involved in urothelial differentiation that are activated by mutations, especially PPARγ and its heterodimerization partner RXRα in the luminal unstable and ELF3 in the luminal unspecified subtype. Mutations in *FOXA1* may contribute. However, despite activation of urothelial differentiation, these tumor cells do not exit the cell cycle. This might be explained partly by the loss of TP53 and especially RB1 inactivation, which prevent cell cycle exit and terminal differentiation. Loss of cell cycle control in general and RB1 activity specifically may account for the overexpression of many genes involved in DNA replication and cell cycle progression as revealed by our analysis (Figure 1B). Moreover, oncogenic PPARγ may activate additional growth signals, such as the SHH pathway [43]. It is unclear how loss of the trithorax-like factors contributes to the phenotype of these other luminal subtypes. Notably, lineage-specific oncogenes in other cancers require functional co-activators. For instance, KDM6A, KMT2C, and the SWI/SNF complex support estrogen receptor activity in luminal breast cancers, although these chromatin regulators may be inactivated during further progression and development of resistance to anti-estrogenic treatments (reviewed in [149]).

In contrast, the BASQ and neuronal-like transdifferentiated UC subtypes do not express markers of urothelial differentiation. Whereas FOXA1, GATA3, and PPARγ support expression of luminal subtype-specific genes, TP63, STAT3, and TFAP2 are main determinators of the basal cell phenotype [150]. Accordingly, transcription factors directing luminal differentiation are often repressed in BASQ cancers [151,152]. The retention of a basal state in BASQ UC could be explained by a predominance of PRC2 polycomb activity if trithorax-like factors driving differentiation are mutated. PRC2 would then maintain a proliferative basal cell precursor phenotype, as demonstrated in epidermal cells [129]. Notably, the histone demethylase KDM1A/LSD1, with dual specificity for H3K4 and H3K9, likewise represses key transcription factors for keratinocyte fate and differentiation [153]. Inhibition of LSD1 conversely induces terminal epidermal differentiation via NOTCH3, ZNF750, and the Grainyhead transcription factors GRHL1 and GRHL3 [154]. The function of KDM1A in urothelial differentiation and UC is however poorly understood [155]. In any case, enhanced EGFR signaling may provide one stimulus for the continuous proliferation of the BASQ subtype cells [39,156], particularly in the absence of functional TP53 and RB1.

Another characteristic of the BASQ subtype is its plasticity, which results in more or less extensive squamous differentiation. Plasticity with loss of lineage fidelity is a frequent phenomenon in epithelial cells proliferating during wound healing and proliferation [157]. It is observed even in cultured normal urothelial cells, which can be stimulated to undergo either squamous or urothelial differentiation, depending on the stimulus [34,158]. It further manifests as benign squamous metaplasia in urinary bladders, often associated with chronic inflammation [159]. As in other instances of squamous metaplasia, in the bladder, it may constitute an adaptive response to protect epithelia from noxious conditions [160]. In UC, clearly, loss of the ability for urothelial differentiation does not preclude aberrant differentiation to a squamous phenotype. Again, loss of TP53 and RB1 function appears to favor not only continuous proliferation but also metaplasia.

Loss of these two regulators is also observed in rare sarcomatoid bladder cancers, [161] which are thought to arise from basal cells. It is moreover fundamental for the development of many cancers with a neuroendocrine phenotype, including some androgen-independent prostate cancers and small-cell lung carcinomas [162,163], and evidently neuronal-like UC [39]. To which extent inactivation of trithorax-like chromatin regulators contributes to this type of plasticity is currently not fully understood. Our present analysis (see Section 2) suggests alterations in Co-REST components to be relevant. A prominent physiological function of this co-repressor complex is suppression of non-neuronal lineages in neuronal cells, but in cancers it may exert a number of alternative functions. Its functionality in neuronal-like UC would therefore be of great interest.

Finally, the molecular subtypes differ also in the type and extent of immune cell infiltration. Immune cell infiltration is most pronounced in the luminal-unspecified and the BASQ subtypes [39], whereas the luminal-papillary subtype appears to actively suppress anti-tumor immunity [151,164]. Accumulating evidence implicates chromatin regulators in the regulation of immune responses in UC [165,166,167] and epigenetic drugs, i.e., inhibitors of histone and DNA modifying enzymes, may therefore be used to re-sensitize UC to immunotherapies (reviewed in [168,169,170,171]).

### 3.2. A Model of Urothelial Carcinoma Development

Two recent papers [172,173] have characterized mutations in micro-dissected morphologically normal urothelium obtained from transplant donors or from cystectomies for urothelial carcinoma. Both research groups detected many mutations in normal urothelium, although their frequency was lower than in muscle-invasive UC. Mutant cells were found to expand clonally in normal urothelium, with clones occupying up to several square mm. Some clones in the normal urothelium of UC patients shared mutations with the tumors. In contrast, synchronous carcinoma in situ (CIS) were clearly related to the main tumor, sharing several mutations but having further evolved along a separate trajectory. Thus, the normal urothelium of middle-aged and elderly persons contains clones of cells with mutant genomes. Importantly, these clones likely account for the often-multifocal emergence of urothelial tumors and may underlie their pronounced tendency to recur.

Whereas point mutations were frequently observed, few of the cell clones expanding in the normal urothelium contained gene copy number changes or chromosomal abnormalities that are prominent in high-grade UC and especially CIS. If at all, entire chromosomes or chromosome arms were lost or gained in normal urothelium. This observation further links chromosomal instability to progression towards invasive carcinoma in urothelial carcinogenesis [4].

**Figure 2:**
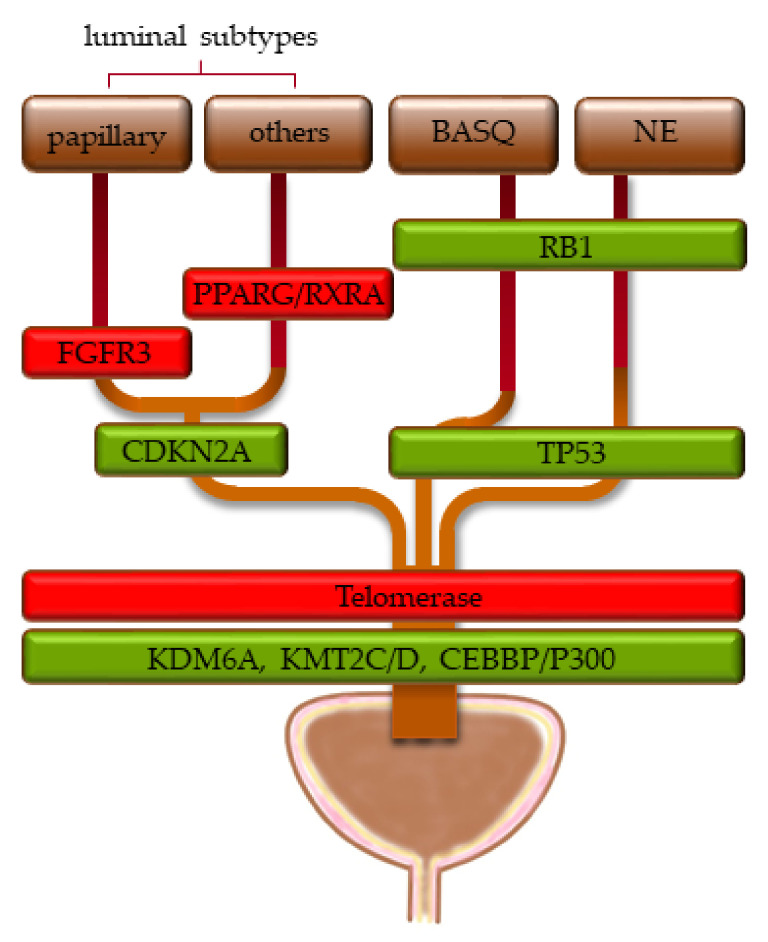
Proposed sequence of genomic changes in urothelial carcinogenesis, proceeding from bottom to top. Green boxes indicate loss of function, red boxes indicate oncogenic activation. The width of the boxes indicates the approximate distribution of the genomic alterations across the molecular subtypes (see Section 1.2 for their definitions).

Intriguingly, in both studies [172,173], chromatin regulator genes were the most frequently mutated functional class of genes, especially *KMT2D*, *KDM6A,* and *ARID1A*, as well as *EP300*, *STAG2,* and *CREBBP*. In contrast, not the activating *FGFR3* mutations observed in most papillary UCs, nor mutations in *TP53* typical of muscle-invasive UC, nor *hTERT* mutations found in ≈80% of UCs across all stages were detected at significant frequencies. These observations suggest that mutations in chromatin regulators occur at an early step of urothelial carcinogenesis and possibly represent an initial—truncal—event. The observation that chromatin regulator mutations are found across all stages and molecular subtypes of UC supports this assumption. Mutations activating proliferative signal transduction pathways and cell cycling, inactivating cellular checkpoints and preventing senescence, would then lead to actual tumor development (Figure 2).

Analogous observations have been made in other tissues (reviewed in [174]). The phenomenon is best characterized in the hematopoietic system, known as clonal hematopoiesis. With increasing age, stem cells in the bone marrow acquire mutations, most often in epigenetic regulator genes (in this case, *DNMT3A*, *TET2*, *ASXL1*). These mutant stem cells expand and replace normal stem cells. While the hematopoietic system remains largely functional, clonal hematopoiesis increases the risk for leukemia. The transformation to leukemia requires additional mutations that activate oncogenes (like *FLT3* or *RAS*) or inactivate tumor suppressors (like *TP53* or *CEBPA*) [175]. Interestingly, specifically *KMT2C* inactivating mutations have been shown to enhance the self-renewal capacity of hematopoietic stem cells [176].

Accordingly, there is no evidence that the mutations in chromatin regulator genes detected in normal urothelium severely affect its functionality or grossly impede its differentiation [172,173]. Moreover, chromatin regulator mutations are found in all molecular subtypes of UC, not only in subtypes like BASQ and neuronal-like lacking markers of urothelial differentiation. This suggests that their primary effect is not to completely block urothelial differentiation, despite the well-established requirement for trithorax-like factors in reprogramming of enhancers and gene expression during development and cell differentiation. Rather, chromatin regulator mutations may enhance the self-renewal ability of urothelial precursor cells, favoring their clonal expansion. In support of this idea, we observed that knockdown of KDM6A in primary urothelial cell cultures and the normal urothelial cell line HBLAK, which comprise basal cells and KRT14-expressing stem cells, increased the proportion of cells expressing KRT14 [148]. Mutations inactivating KDM6A in normal urothelium may therefore augment the capacity of urothelial KRT14-expressing precursor cells for self-renewal, allowing them to gradually colonize the tissue at the expense of non-mutant urothelial precursors. Mutations inactivating other chromatin regulators like KMT2D may act in a similar fashion. Evidently, more experimental evidence is required to decide whether this hypothesis applies to urothelial carcinogenesis.

Several questions in particular need to be addressed to validate and clarify this hypothesis. First, by which mechanisms does inactivation of chromatin regulators enhance self-renewal of urothelial stem cells? Second, in which respect do KRT14-positive stem cells with mutant chromatin regulators differ from normal ones? While not grossly disturbed, to which extent is their differentiation ability impeded in detail? Third, do all chromatin regulator mutations favor carcinogenesis to the same extent? Fourth, do they favor the development of specific molecular subtypes? For instance, *KDM6A* mutations appear to be particularly frequent in the luminal-papillary subtype, both in muscle-invasive UC and non-invasive urothelial tumors, and are often accompanied by *FGFR3* mutations. The study by Barrows et al. [144] provides a partial explanation for this association. Similarly, the oncogenic function of mutant RXRA/PPARγ in organoids depended on inactivation of KDM6A as well as TP53 [177]. Moreover, PPARγ was recently demonstrated to induce tumors in basal precursors only following injury, again suggesting that its oncogenic action depends on an altered epigenetic state [152].

The ideas on the function of chromatin regulator mutations in urothelial carcinogenesis outlined above have important implications for targeted therapeutic approaches. Targeted therapies have been relatively unsuccessful in UC, with the recent exception of FGFR inhibitors in the papillary-luminal subtype [41,42]. On this background, the high prevalence of mutations in chromatin regulators provides a further rationale to explore epigenetic drugs, i.e., compounds that influence histone and DNA modifications [178,179]. Ideally, these drugs would reverse the deficits resulting from chromatin regulator inactivation in UC. Appropriate treatments could limit cellular plasticity and exhaust self-renewing cancer cell populations by forcing them into terminal differentiation [180]. However, if inactivation of chromatin regulators occurs at a very early stage of carcinogenesis, it may only set the stage for subsequent transformation and might not necessarily be required for growth and survival of the actual cancers. This issue clearly calls for deeper investigation. The findings so far suggest that restoration of KDM6A function does affect proliferation of UC cells but usually only over time [132,148,181,182]. These delayed effects could be explained by decreased self-renewal ability of tumor stem cells.

While restoration of its function might come too late to suppress tumor growth, lack of a chromatin regulator may sensitize cancers to the inhibition of different chromatin regulators or other processes that are essential for tumor growth, i.e., it may generate synthetic lethality. For instance, ARID1A-deficient tumors are particularly sensitive to inactivation of its paralog ARID1B, which leads to dissociation of the remaining SWI/SNF complexes with alternative composition [183,184]. In UC, specifically, KDM6A inactivation appears to sensitize tumors towards inhibitors of its antagonist, the histone methyltransferase EZH2 [182]. Similarly, since KMT2C mutations diminish the expression of genes required for homologous recombination DNA repair, they may sensitize deficient UC cells to PARP inhibitors like olaparib [137]. The latter example highlights the point that mutations in chromatin regulators might not only affect cell differentiation but also other cellular processes, including DNA repair, metabolism, and cell adhesion [182,185,186,187,188,189]. Moreover, with an increasing repertoire of immunotherapies, the emerging effects of chromatin regulator deficiencies on anti-tumor immune responses [165,166,167,190,191] could provide new approaches for targeted therapy in UC.

## 4. Conclusions—Prospects

In this review, we re-analyzed data from the TCGA comprehensive investigation of genetic alterations in urothelial carcinoma with respect to chromatin regulators and summarized the state of the literature on their functions in this cancer type. Our data analysis revealed additional candidate factors and relations that merit further investigation in urothelial carcinoma to improve the understanding of pathogenetic mechanisms in this cancer. The literature analysis led us to propose a hypothesis on the biological function of the most frequently inactivated—trithorax-like—chromatin regulators in urothelial carcinogenesis. Throughout these analyses, we could not help noting that despite much recent progress, bladder cancer remains under-researched. In particular, mechanistic studies addressing the molecular and cellular consequences of chromatin regulator alterations, specifically in urothelial cells, are sparse. Hopefully, the current review will not only alert to that fact, but provide information and ideas that help to ameliorate these deficiencies.

## Figures and Tables

**Table 1:** Top 25 chromatin regulator genes affected by mutations in urothelial carcinoma.

HGNC Gene Symbol	HGNC Gene Name	Function	Sample Number with Mutations	Cytoband
*TP53*	tumor protein p53	Histone modification write cofactor, TF	230	17p13.1
*KMT2D*	lysine (K)-specific methyltransferase 2D	Histone modification write	160	12q13.12
*ARID1A*	AT rich interactive domain 1A (SWI-like)	Chromatin remodeling cofactor	130	1p36.11
*KDM6A*	lysine (K)-specific demethylase 6A	Histone modification erase	117	Xp11.3
*KMT2C*	lysine (K)-specific methyltransferase 2C	Histone modification write	103	7q36.1
*RB1*	retinoblastoma 1	Chromatin remodeling, Histone modification write	83	13q14.2
*EP300*	E1A binding protein p300	Histone modification write	80	22q13.2
*ATM*	ATM serine/threonine kinase	Histone modification write	71	11q22.3
*KMT2A*	lysine (K)-specific methyltransferase 2A	Histone modification write	59	11q23.3
*CREBBP*	CREB binding protein	Histone modification write	53	16p13.3
*BRCA2*	breast cancer 2, early onset	Histone modification write	52	13q13.1
*ASXL2*	additional sex combs like transcriptional regulator 2	Histone modification read	47	2p23.3
*NCOR1*	nuclear receptor corepressor 1	Histone modification erase cofactor	44	17p12-p11.2
*TRRAP*	transformation/transcription domain-associated protein	Histone modification write cofactor	41	7q22.1
*SRCAP*	Snf2-related CREBBP activator protein	Chromatin remodeling, Histone modification erase	38	16p11.2
*CHD7*	chromodomain helicase DNA binding protein 7	Chromatin remodeling	37	8q12.2
*MGA*	MGA, MAX dimerization protein	Histone modification write cofactor, TF	36	15q15.1
*ATR*	ATR serine/threonine kinase	Histone modification write	36	3q23
*ASH1L*	ash1 (absent, small, or homeotic)-like (Drosophila)	Histone modification write	36	1q22
*PRKDC*	protein kinase, DNA-activated, catalytic polypeptide	Histone modification write	35	8q11.21
*CHD6*	chromodomain helicase DNA binding protein 6	Chromatin remodeling	35	20q12
*SETD2*	SET domain containing 2	Histone modification write	33	3p21.31
*CHD2*	chromodomain helicase DNA binding protein 2	Chromatin remodeling	33	15q26.1
*ARID1B*	AT rich interactive domain 1B (SWI1-like)	Histone modification write	32	6q25.3
*SPEN*	spen family transcriptional repressor	Histone modification erase cofactor, TF	32	1p36.21-p36.13

**Table 2:** Top 25 chromatin regulator genes amplified in urothelial carcinoma.

HGNC Gene Symbol	HGNC Gene Name	Function	Sample Number with Amplifications	Cytoband
*USP21*	ubiquitin specific peptidase 21	Histone modification erase	69	1q23.3
*YWHAZ*	tyrosine 3-monooxygenase/tryptophan 5-monooxygenase activation protein, zeta	Histone modification read	69	8q22.3
*UBR5*	ubiquitin protein ligase E3 component n-recognin 5	Chromatin remodeling, Histone modification write cofactor	58	8q22.3
*TADA1*	transcriptional adaptor 1	Histone chaperone	46	1q24.1
*SETDB1*	SET domain, bifurcated 1	Histone modification write	44	1q21.3
*BRD9*	bromodomain containing 9	Histone modification read	42	5p15.33
*TAF3*	TAF3 RNA polymerase II, TATA box binding protein (TBP)-associated factor, 140 kDa	Histone modification read	41	10p14
*CHD1L*	chromodomain helicase DNA binding protein 1-like	Chromatin remodeling	40	1q21.1
*PRKAB2*	protein kinase, AMP-activated, beta 2 non-catalytic subunit	Histone modification write cofactor	40	1q21.1
*VPS72*	vacuolar protein sorting 72 homolog (S. cerevisiae)	Histone modification write cofactor	40	1q21.3
*ZNF687*	zinc finger protein 687	Histone modification erase cofactor	40	1q21.3
*POGZ*	pogo transposable element with ZNF domain	Histone modification read	39	1q21.3
*ANP32E*	acidic (leucine-rich) nuclear phosphoprotein 32 family, member E	Histone chaperone, Histone modification read	38	1q21.2
*TDRKH*	tudor and KH domain containing	RNA modification	38	1q21.3
*SETD5*	SET domain containing 5	Histone modification write	38	3p25.3
*ASH2L*	ash2 (absent, small, or homeotic)-like (Drosophila)	Histone modification write cofactor	38	8p11.23
*HDAC11*	histone deacetylase 11	Histone modification erase	37	3p25.1
*BRPF1*	bromodomain and PHD finger containing, 1	Histone modification read	37	3p25.3
*TADA3*	transcriptional adaptor 3	Histone modification write cofactor	37	3p25.3
*ATAD2*	ATPase family, AAA domain containing 2	Chromatin remodeling	35	8q24.13
*ZHX1*	zinc fingers and homeoboxes 1	Chromatin remodeling, Histone modification write cofactor	35	8q24.13
*SFMBT2*	Scm-like with four mbt domains 2	Histone modification read, Polycomb group (PcG) protein, TF	35	10p14
*NIPBL*	Nipped-B homolog (Drosophila)	Histone modification erase cofactor	33	5p13.2
*YEATS4*	YEATS domain containing 4	Histone modification write cofactor	33	12q15
*RAD54B*	RAD54 homolog B (S. cerevisiae)	Chromatin remodeling	32	8q22.1

**Table 3:** Top 25 chromatin regulator genes affected by homozygous deletions in urothelial carcinoma.

HGNC Gene Symbol	HGNC Gene Name	Function	Sample Number with Homozygous Deletions	Cytoband
*RB1*	retinoblastoma 1	Chromatin remodeling, Histone modification write	37	13q14.2
*USP17L2*	ubiquitin specific peptidase 17-like family member 2	Histone modification erase cofactor	31	8p23.1
*NPM2*	Nucleophosmin nucleoplasmin 2	Histone chaperone	30	8p21.3
*HR*	hair growth associated	Histone modification erase	29	8p21.3
*SETDB2*	SET domain, bifurcated 2	Histone modification write	29	13q14.2
*ELP3*	elongator acetyltransferase complex subunit 3	Histone modification write	24	8p21.1
*PBK*	PDZ binding kinase	Histone modification write	24	8p21.1
*ERBB4*	v-erb-b2 avian erythroblastic leukemia viral oncogene homolog 4	Histone modification cofactor	20	2q34
*PSIP1*	PC4 and SFRS1 interacting protein 1	Chromatin remodeling	17	9p22.3
*FOXO1*	forkhead box O1	TF	17	13q14.11
*ING5*	inhibitor of growth family, member 5	Histone modification read	16	2q37.3
*JAK2*	Janus kinase 2	Histone modification write	16	9p24.1
*KDM4C*	lysine (K)-specific demethylase 4C	Histone modification erase	16	9p24.1
*UHRF2*	ubiquitin-like with PHD and ring finger domains 2, E3 ubiquitin protein ligase	Histone modification read	16	9p24.1
*TDRD3*	tudor domain containing 3	Histone modification read	15	13q21.2
*HDAC4*	histone deacetylase 4	Histone modification erase	14	2q37.3
*EXOSC8*	exosome component 8	Scaffold protein, RNA modification	14	13q13.3
*CREBBP*	CREB binding protein	Histone modification write	14	16p13.3
*NCOR1*	nuclear receptor corepressor 1	Histone modification erase cofactor	14	17p12-p11.2
*CUL3*	cullin 3	Histone modification write	13	2q36.2
*SP140*	SP140 nuclear body protein	Histone modification read, TF	13	2q37.1
*FOXP1*	forkhead box P1	TF	13	3p13
*CTBP2*	C-terminal binding protein 2	Histone modification write cofactor	12	10q26.13
*TRIM16*	tripartite motif containing 16	Histone modification write	12	17p12
*HJURP*	Holliday junction recognition protein	Histone chaperone	11	2q37.1

## Data Availability

The analyzed data are publicly available from the Morpheus database or Supplementary Materials of ref. [7]. All results of data analyses presented in this study are available in Supplementary Data File 1.

## Supplementary Materials

The following are available online at https://www.mdpi.com/article/10.3390/cancers13236040/s1, Data file S1: Complete data for genomic alterations and significant expression changes of chromatin regulators.
